# An outbreak associated with *Escherichia albertii* in a junior high school, China

**DOI:** 10.1017/S0950268824001341

**Published:** 2024-10-04

**Authors:** Shiwang Huang, Qian Liu, Yezhen Fang, Hua Yu, Xi Yang, Jinfeng Hu, Yiyi Wang, Rui Tian, Yixiao Gao, Zhimin Ni, Yanwen Xiong

**Affiliations:** 1 Laboratory of Microbiology, Shangcheng District Center for Disease Control and Prevention, Hangzhou, China; 2 National Key Laboratory of Intelligent Tracking and Forecasting for Infectious Diseases, National Institute for Communicable Disease Control and Prevention, Chinese Center for Disease Control and Prevention, Beijing, China; 3 Laboratory of Microbiology, Hangzhou Municipal Center for Disease Control and Prevention, Hangzhou, China

**Keywords:** *Escherichia albertii*, foodborne-pathogen, gastroenteritis, outbreaks, whole-genome sequencing

## Abstract

*Escherichia albertii* is an emerging foodborne enteropathogen associated with infectious diarrhoea in humans. In February 2023, an outbreak of acute gastroenteric cases was reported in a junior high school located in Hangzhou, Zhejiang province, China. Twenty-two investigated patients presented diarrhoea (22/22, 100%), abdominal pain (21/22, 95.5%), nausea (6/22, 27.3%), and vomiting (3/22, 13.6%). *E. albertii* strains were successfully isolated from anal swabs collected from six patients. Each isolate was classified as sequence type ST2686, harboured *eae*-β gene, and carried both *cdtB*-I and *cdtB*-II subtypes, being serotyped as EAOg32:EAHg4 serotype. A comprehensive whole-genome phylogenetic analysis revealed that the six isolates formed a distinct cluster, separate from other strains. These isolates exhibited minimal genetic variation, differing from one another by 0 to 1 single nucleotide polymorphism, suggesting a common origin from a single clone. To the best of our knowledge, this represented the first reported outbreak of gastroenteritis attributed to *E. albertii* outside of Japan on a global scale.

*Escherichia albertii*, a bacterium closely related to *E. coli*, is an emerging enteropathogen causing sporadic infectious diarrhoea and gastroenteric outbreaks in humans. It has often been misidentified as enteropathogenic or enterohemorrhagic *E. coli* (EPEC or EHEC), leading to the underestimation of *E. albertii* infections [1]. *E. albertii* infections typically cause watery diarrhoea, abdominal pain, and fever, with most cases resolving without complications [1]. The presence of a type III secretion system encoded by the locus of enterocyte effacement (LEE), cytolethal distending toxin (CDT), Shiga toxins, and other virulence factors contribute to clinical manifestations of this pathogen [2].

From 14 to 16 February 2023, an outbreak of diarrhoea was reported at a junior high school in Hangzhou, Zhejiang province, China. A suspected case was defined as those experiencing three or more episodes of diarrhoea with or without vomiting within 24 h from 13 to 16 February in this school. Based on the case definition, a total of 22 out of 770 students were defined as suspected cases, giving an overall morbidity rate of 2.9% (22/770). The major symptoms were diarrhoea (22/22, 100%), abdominal pain (21/22, 95.5%), nausea (6/22, 27.3%), and vomiting (3/22, 13.6%). No fevers were reported among the cases. According to the epidemiological investigation, all 770 students were in the third grade and had the same dinner on 13 February from a catering delivery company. Unfortunately, no food samples were kept for analysis on 13 February. The first case occurred at 17:00 on 14 February with the last case being reported at 12:00 on 15 February. A peak in the incidence curve was observed between 19:00 and 21:00 on 14 February, making a duration of approximately 19 h from the onset of the first to the last case. The suspicious meal was consumed at 17:00 on 13 February, indicating that the incubation period for the *E. albertii* infection causing this outbreak ranged from 24 to 43 h.

Anal swabs from seven patients presenting with diarrhoea were collected, pooled, and screened for enteropathogens using FilmArray™ gastrointestinal (GI) panel (MEP, BioFire Diagnostics/Biomerieux, Salt Lake City, UT, USA). The initial screening identified the presence of *eae* gene exclusively in the pooled samples. To further investigate, nucleic acid was extracted from seven anal swabs, complemented by 16 environment smears – comprising ten samples from desk surfaces, four from the interiors of refrigerators, and two from water dispenser outlets – and 12 food samples supplied on 14–15 February. These were subsequently analysed using the Multiplex Real-Time PCR Diagnostic Kit for Rapid Identification of Diarrhoeagenic *Escherichia coli* (XABT, Beijing, China). The analysis revealed that six anal swabs samples and one desk surface smear tested positive for both *eae* and *uidA* genes. The *uidA* gene, which encodes the beta-glucuronidase enzyme, was a common marker in both commensal and pathogenic (diarrhoeagenic) *E. coli* strains and served as a reference gene in the polymerase chain reaction (PCR) diagnostic process [3]. According to the kit’s guidelines, a strain was classified as diarrhoeagenic *E. coli* if it tested positive for the *uidA* gene along with at least one additional virulence gene. In contrast, strains positive solely for the *uidA* gene were categorised as commensal or non-pathogenic *E. coli.* All *eae*-positive samples were inoculated onto CHROMagar^TM^ ECC agar (CHROMagar, Paris, France) and incubated overnight at 37°C. The colourless colonies, isolated from six patients’ samples, were *eae*-positive but *uidA*-negative. The presumptive colonies were non-motile, negative for fermentation of lactose, xylose, sucrose, rhamnose, and melibiose, and absent of indole and tryptophan decarboxylase which were determined by using bacterial biochemical identification tube (Hopebiol, Qingdao, China). The colonies were further identified as *E. albertii* by using diagnostic triplex-PCR targeting *clpX*, *lysP*, and *mdh* genes [4]. To confirm the adherence patterns of isolates, HEp-2 cell adherence assay was performed as previously described [5] with minor modifications. Briefly, monolayers of 10^5^ HEp-2 cells were grown in Dulbecco’s modified Eagle medium containing 10% foetal bovine serum on 24-well tissue culture plates. Bacterial strains were grown in 5 mL of Luria-Bertani (LB) broth at 37°C with shaking at 180 rpm for 2–3 h to reach an optical density of 0.5 at 600 nm. Cell monolayers were infected with bacterial cultures at a multiplicity of infection (MOI) of 1:100. After a 6 h incubation period at 37°C, the cells were washed with sterile PBS, fixed with methanol, stained with Giemsa solution, and examined under a light microscope. The six isolates showed localised adherence to cultured HEp-2 cells. Furthermore, the susceptibility testing of these isolates to 26 antimicrobials, conducted using VITEK^®^ 2 AST-N334 and AST-GN09 (bioMérieux, Marcy-l’Étoile, France), demonstrated that all were sensitive to the tested antimicrobials.

The total DNA of isolates was extracted using the Wizard® Genomic DNA Purification Kit (Promega, Madison, WI, USA). Fragment libraries of the genomic DNA were generated using the Universal DNAseq Library Prep Kit (Kaitai-Bio, Hangzhou, China) and sequenced on the Illumina HiSeq 2000 platform (Illumina, San Diego, CA, USA). *De novo* assembly and genomic assessment were performed using Unicycler v0.4.8 and QUAST v5.2.0, respectively, as previously described [4]. The raw sequencing reads obtained in this study have been archived in the National Centre for Biotechnology Information (NCBI) under BioProject accession PRJNA993394.

The multi-locus sequence types (STs) of isolates were determined using the PubMLST online platform (https://pubmlst.org/organisms/escherichia-spp). The *eae* and *cdtB* subtypes of isolates were identified by ABRicate v1.0.1 (https://github.com/tseemann/abricate.git) with sequence coverage of 70% and identity of 97%. Briefly, local subtyping databases were complied with ABRicate, integrating established nucleotide sequences for all recognized *eae* and *cdtB* subtypes, as previously reported [6]. The assemblies were then analysed against these subtyping databases. The *E. albertii* O- and H-antigen genotypes (EAOg/EAHg) were determined as previously described [4]. The presence of virulence and antibiotic-resistant genes were identified using the ABRicate against Virulence Finder database (VFDB) and Resfinder database with default parameters, respectively. Results showed that all six isolates in this study were classified as ST2686, carried *eae*-β, *cdtB*-I, and *cdtB*-II subtypes, and were serotyped as EAOg32:EAHg4 (Figure 1). The macrolide-associated resistance gene *mdf(A)* and the K88 pili/F4 fimbriae-related genes (*faeC/E/F/H/I/J*) were detected in all six isolates. The enterotoxin (*entA/B/C/D/E/F/S*, *fepA/B/C/D/G*, and *fes*), type 1 fimbriae (*fimA/B/C/D/E/F/G/H/I*), and type II secretion system (*gspC/D/E/F/G/H/I/J/K/L/M*) related genes, commonly present in *E. albertii* strains, were also identified in these isolates. All virulence genes were full length with no premature stop codons.

**Figure 1.**
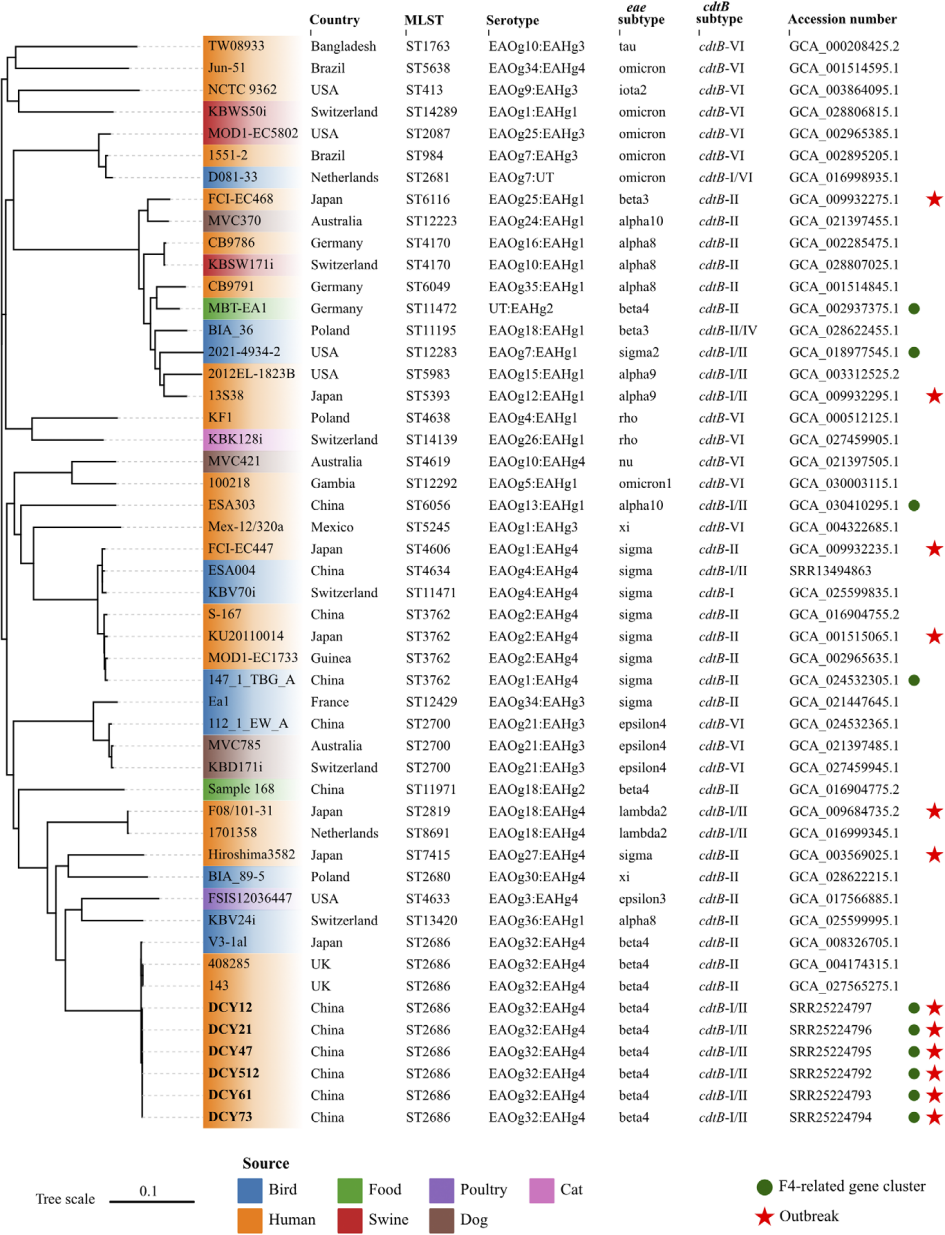
Whole-genome phylogenetic tree based on single nucleotide polymorphisms. Forty-three *E. albertii* genomes were retrieved from the NCBI database. Six strains sequenced in this study were indicated in bold. *E. albertii* S-167 was used as the reference strain. The colours of leaves represent different sources of strains. The red stars indicate the strains isolated from outbreaks. The green circles represent the presence of the F4-related gene cluster. The scale represents the number of substitutions per site.

To assess the phylogenetic relationships among outbreak strains in this study and other *E. albertii* strains, we retrieved 43 publicly available *E. albertii* genomes from the NCBI database, complemented by all available epidemiological information. The 43 strains were isolated from different sources in 13 countries between 1983 and 2022, including three strains with the same ST2686 and six strains associated with previous gastroenteritis outbreaks. Snippy v3.2 (https://github.com/tseemann/snippy.git) was used to map genomes to the *E. albertii* reference strain S-167 (GCA_016904755.1) with default parameters. FastTree v2.0 was used to generate an approximate maximum likelihood phylogenomic tree based on the general time reversible model [7]. Subsequently, the core alignment output from snippy was applied to calculate the single nucleotide polymorphisms (SNP) distance between isolates using snp-dists v0.8.2 (https://github.com/tseemann/snp-dists) with default parameters. The six isolates from this study coalesced into a single, highly genetically related cluster, with a minimal genetic divergence ranging from 0 to 1 SNP. This genetic homogeneity implied a common clonal origin for the isolates, which was responsible for the GI outbreak under investigation. To the best of our knowledge, this represented the first reported outbreak of gastroenteritis caused by *E. albertii* outside of Japan on a global scale. The isolates in this study showed a close genetic relationship with two human-derived strains from UK and one bird-derived strain from Japan, with SNPs distance of 42, 42, and 34, respectively (Figure 1). However, they were genetically discrete from strains associated with six gastroenteritis outbreaks in Japan. These results suggested that no known or dominant *E. albertii* types are predictive for outbreaks, and strains from multiple sources can potentially cause outbreaks.

*E. albertii* is an emerging enteropathogen widely present in poultry, birds, and raw meats in China [6]. However, its prevalence in humans is relatively low [8]. Six outbreaks caused by *E. albertii* have been reported, which were mostly associated with contaminated water or food [9]. In this study, the morbidity rate associated with *E. albertii* outbreak was significantly lower than those reported in previous studies [9]. This discrepancy may be attributed to the early intervention measures taken at the onset of the outbreak, where classes were suspended and students were sent home, with only individuals exhibiting severe symptoms being enrolled in the study, potentially leading to an underestimation of the true morbidity rate.

In addition, the epidemiological investigation revealed that the school consists of the second and third grades, with students from both grades sharing the same drinking water source. However, meals for second- and third-year students were delivered by two different food delivery companies. Notably, only third-grade students had been affected by the outbreak. Thus, although *E. albertii* was not detected in the limited food samples in this study, the contaminated food was considered as the most probable vehicle for this outbreak.

Interestingly, six isolates were found to carry the K88 (F4) fimbriae adhesin genes, which have been identified in enterotoxigenic *E. coli* (ETEC) responsible for significant morbidity and mortality in newborn and weaned piglets [10]. Various fimbriae enable bacteria to adhere closely to, colonize, or invade host cells, thereby persisting and thriving within the localised host environment, which leads to disease development. The potential involvement of the F4-related gene cluster in *E. albertii* needs to be explored.

## Data Availability

Raw sequencing reads of *E. albertii* isolates were deposited in NCBI under the Bioproject number PRJNA993394.
